# Defeat Dengue and Zika Viruses With a One-Two Punch of Vaccine and Vector Blockade

**DOI:** 10.3389/fmicb.2020.00362

**Published:** 2020-03-20

**Authors:** Jin Sun, Senyan Du, Zhihang Zheng, Gong Cheng, Xia Jin

**Affiliations:** ^1^Viral Disease and Vaccine Translational Research Unit, CAS Key Laboratory of Molecular Virology and Immunology, Institut Pasteur of Shanghai, Chinese Academy of Sciences, Shanghai, China; ^2^Tsinghua-Peking Center for Life Sciences, School of Medicine, Tsinghua University, Beijing, China; ^3^Institut Pasteur of Shanghai, Chinese Academy of Sciences, University of Chinese Academy of Sciences, Shanghai, China; ^4^Shanghai Public Health Clinical Center, Fudan University, Shanghai, China

**Keywords:** dengue virus, Zika virus, epidemiology, virology, protective immunity, vaccine, mosquitoes

## Abstract

Dengue virus (DENV) and Zika virus (ZIKV) are two mosquito-borne flaviviruses afflicting nearly half of the world population. Human infection by these viruses can either be asymptomatic or manifest as clinical diseases from mild to severe. Despite more cases are presented as self-limiting febrile illness, severe dengue disease can be manifested as hemorrhagic fever and hemorrhagic shock syndrome, and ZIKV infection has been linked to increased incidence of peripheral neuropathy Guillain-Barre syndrome and central neural disease such as microcephaly. The current prevention and treatment of these infectious diseases are either non-satisfactory or entirely lacking. Because DENV and ZIKV have much similarities in genomic and structural features, almost identical mode of mosquito-mediated transmission, and probably the same pattern of host innate and adaptive immunity toward them, it is reasonable and often desirable to investigate these two viruses side-by-side, and thereby devise common countermeasures against both. Here, we review the existing knowledge on DENV and ZIKV regarding epidemiology, molecular virology, protective immunity and vaccine development, discuss recent new discoveries on the functions of flavivirus NS1 protein in viral pathogenesis and transmission, and propose a one-two punch strategy using vaccine and vector blockade to overcome antibody-dependent enhancement and defeat Dengue and Zika viruses.

## Epidemiology of Dengue Virus and Zika Virus

Dengue virus (DENV) is the most prevalent mosquito-borne flavivirus that affects over half of the world population in 128 countries and regions (Brady et al., 2012). Zika virus (ZIKV) is another virus that has caught public attention while sweeping across 60 countries and infecting several million people during 2014–2016 (Baud et al., 2017). The transmitting vectors of DENV and ZIKV are *Aedes* mosquitoes, mainly *A. aegypti* and *A. albopictus* (Guzman et al., 2010; Weaver et al., 2016). These viruses cause human diseases that share some similar clinical manifestations but also have features that are distinct from each other.

Dengue viruses are antigenically classified into four serotypes that originate from one ancestor phylogenetically and cause common pathologies in human. Infection by any serotype of dengue virus can be either symptomatic (∼25%) or not (Bhatt et al., 2013; St John and Rathore, 2019). The majority of symptomatic infection only develop self-limited febrile illness with manifestations of high fever, facial flushing, rash, myalgia, arthralgia, headache, retro-orbital pain, vomiting, epistaxis, or gum bleeding. Febrile phase lasts 2–7 days, during which viremia and plasma NS1 can be detected in the initial 1–4 days. Only a small percentage of the symptomatic patients (mostly children) progress to more severe forms of dengue disease, Dengue Hemorrhagic Fever (DHF) and Dengue Shock Syndrome (DSS), in which plasma leakage and hemorrhagic manifestations occur; meanwhile, leukopenia, hemoconcentration, and thrombocytopenia can be detected by laboratory tests. Severe plasma leakage that leads to metabolic acidosis and shock with occasional organ impairment is fetal without proper treatment (World Health Organization [WHO], 2009). Immune responses induced during infection by one serotype of dengue virus can confer life-long protection against homotypic viruses and provide cross-protection toward heterotypic viruses for short-term. Strikingly, such cross-reactivities have the potential to enhance disease severity of a secondary heterotypic infection, probably through a mechanism called Antibody Dependent Enhancement (ADE). Consistently, increased risk of DHF/DSS has been observed for secondary dengue virus infections in some epidemiological studies (Sangkawibha et al., 1984; Guzman et al., 2013; Katzelnick et al., 2017). Dengue like disease has been a public health problem for over two centuries. In 1780, Benjamin Rush recorded in Philadelphia an epidemic of bilious remitting fever, which is now considered as one of the earliest descriptions of dengue like diseases in America (Rush, 1951; Packard, 2016). In the following decades, such outbreaks appeared throughout Atlantic World. The name “dengue virus” appeared in the Caribbean in 1820s, it might have derived from Swahili “*Ka-dinga-pepo*” or Spanish “*denguero*,” meaning seizures caused by an evil spirit or referring to the special gait, to describe the agonizing status after infection (Barnett, 2017). Nevertheless, whether all of these early epidemics were caused by dengue virus rather than another clinically similar arbovirus, chikungunya virus, was uncertain, as disease of these outbreaks was often named as “break bone fever” and some were described as rheumatic diseases (Carey, 1971). Although it is unclear when dengue virus was first introduced to Asia, as sylvatic strains of DENV-1 (strain P72-1244), -2 (strain P8-1407), -4 (strain P73-1120 and P75-514, P75-215) were isolated from sentinel monkeys or *A. niveus* mosquitoes in the rainforest of Malaysia, and the existence of sylvatic DENV-3 is supported indirectly by monkey seroconversion against DENV-3 in Malaysia, it is suggested that the four serotype viruses evolved independently in the rainforests of southeast Asia based on the phylogenic analysis (Wang et al., 2000; Holmes and Twiddy, 2003). The huge troop deployments during the Second World War might have also exacerbated the spread of dengue viruses in Asia (Clarke, 2002). Since the beginning of twentieth century, due to the increased urbanization and lacking control of *Aedes* mosquitoes, all four serotypes of dengue viruses have been found in South Asia, causing endemics each year. Similarly, multiple serotypes of dengue virus were circulating in tropical and sub-tropical regions of America after 1950s, leading to regular outbreaks in these regions (Gubler, 1987). Coincide with this, increased DHF and DSS cases were reported in different outbreaks after World War II (Halstead, 1966; World Health Organization [WHO], 1966). Now, the four serotype viruses are co-circulating in all endemic regions (Mackenzie et al., 2004), and estimated to infect 390 million people, causing 10,000 death, and 96 million symptomatic infections in 128 countries each year (Guzman et al., 2010; Brady et al., 2012; Bhatt et al., 2013). Roughly, the world population live under the threat of dengue disease is recently predicted to increase to over 60% by 2080 (Messina et al., 2019). Although modern era of dengue research has begun since Sabin and his colleagues first isolated the virus in 1944 (Sabin, 1952), the dissemination of dengue viruses are still continuing, as no antiviral drugs are available and the only licensed vaccine is far from satisfactory.

In comparison, Zika virus emerged more recently as a public health concern. The historical documents of Zika virus started as its first isolation from a monkey in Zika forest of Uganda in 1947 (Dick et al., 1952). In the following decades, only a few sporadic outbreaks were reported in Africa and South Asia, the number of cases during each outbreak never exceeded a few dozen before 2007 (Duffy et al., 2009; Grard et al., 2014). Symptoms of ZIKV infection including fever, arthralgia, rash and conjunctivitis were presented in patients during the early outbreaks, although 50–80% infections were asymptomatic (Weaver et al., 2016). Fundamental epidemic and clinical changes of the disease first appeared in an outbreak in French Polynesia in 2013, when 28,000 people were infected, and Guillain-Barre syndrome was observed for the first time to be associated with Zika virus infection, implicating the neurotropic feature of Zika virus (Musso et al., 2018); additionally, detection of viral RNA in semen and urine were also documented during this outbreak. Nevertheless, the threat of Zika virus was not recognized until 2015–2016 when it caused much larger outbreaks in South America, most pronounced in Brazil (Campos et al., 2015). In this outbreak, 40% notified Brazilian cases were from northeast region of Brazil (Faria et al., 2017), where Zika virus infected 63% peoples in Salvador. The virus spread to most countries in Americas (Netto et al., 2017), and then further expanded into a pandemic with several outbreaks in South Asia and pacific islands (Baud et al., 2017). In Europe, travel-associated ZIKV cases were reported in most EU (European Union) countries during 2015–2016, with the largest number of cases in France, and largest percentage related to the Caribbean (Spiteri et al., 2017). During the Brazil outbreak, ZIKV has been suspected to persistently replicate in multiple organs for months, and confirmed to be capable of sexual and vertical transmission; and found to be associated with increased ratios of microcephaly and congenital diseases in newborn babies (Weaver et al., 2016). Soon after, the association between Zika virus infection and microcephaly was confirmed by extensive studies in mice (Li et al., 2016; Yockey et al., 2016; Vermillion et al., 2017; Szaba et al., 2018). Furthermore, *in vivo* replication of ZIKV in neuron, placenta and fetus was also verified by using autopsy samples (Martines et al., 2016; Mlakar et al., 2016). Since early 2016, WHO has declared Zika virus disease as a public health emergency of global concern.

It should be noted that ZIKV and DENV spread through the same *Aedes* mosquito vectors in overlapping epidemic areas, and cross-reactivity of their adaptive immune responses are also often observed, therefore, inspecting their similarity in viral and genomic structures should be of value.

## Virological Features of Dengue Virus and Zika Virus

Both Dengue virus and Zika virus belong to the genus of *flavivirus* in the *flaviviridae* family, and they have similar genome organization and virion morphology. They both have one single-stranded positive RNA as genome, which contain an open reading frame (ORF) of ∼11,000 nucleotides, flanked by 100 nucleotides at the 5′UTR and 400–500 nucleotides at the 3′UTR. The single ORF encodes a polyprotein of 3000-amino acid that is translationally or post-translationally processed into three structural proteins (C, prM, E) and seven non-structural proteins (NS1, NS2A, NS2B, NS3, NS4A, NS5). Generally, the sequence homology between DENV and ZIKV polyprotein is 55–56%, whereas the homology among four serotype dengue virus polyproteins is 69–72%. The three structural proteins and phospholipid bilayers derived from ER of infected cells form the major architecture of viral particles of ZIKV and DENV, whereas the non-structural proteins (except for NS1) mainly retain intracellular location and participate in viral RNA replication and viral polyprotein processing: NS5 is RNA dependent RNA polymerase and methyltransferase, NS3 is helicase, NS2B/NS3 complex is a serine protease that processes polyprotein at C/pr, NS2A/NS2B, NS2B/NS3, NS3/NS4A, NS4A/2K, and NS4B/NS5 junctions. The NS proteins also interact with host immunity: NS2B/NS3, NS4A, NS4B, and NS5 can be antagonists of innate immune responses; NS1 can directly or indirectly cause pathology (Lindenbach et al., 2007; Ngono and Shresta, 2018). On the surface of the mature virus particles, 90 copies of E homodimers lie flat on the virus membrane, packed in a herringbone array; each E dimer is comprised of two antiparallel monomers whose c-terminal helixes are anchored into the lipid bilayer of virus envelope. As envelope protein directly mediates receptor binding and post-entry membrane fusion, it is mostly outwardly exposed and thus contains most neutralization epitopes. Underneath each dimeric E ectodomains are a pair of M proteins buried in the pockets and holes formed between two E subunits on the viral membrane. Similarly, M proteins are anchored to membrane through c-terminal tans-membrane helixes, complexed with each E protein and modulating conformational change of E to initiate membrane fusion at post-entry stage in a pH dependent manner (Zhang et al., 2013; Sirohi et al., 2016). Inside the envelope, the capsids interact with genomic RNA, forming a spherical nucleocapsid, in which ordered structure of capsid surrounding RNA has never been observed in dengue viruses; but a recent study at 9 Å resolution showed structure of immature Zika virus and demonstrated that there was a regular but broken shell of capsid inside the membrane, implying a dynamical morphological alteration during Zika virus maturation (Prasad et al., 2017). Despite minor differences, the structures of DENV and ZIKV are generally similar. A noted difference exists in a region surrounding glycosylation site at Asn^154^ of ZIKV envelope protein and Asn^153^ of DENV envelope protein, these sites may influence virus transmission and pathology through affecting receptor binding and cell tropism (Sirohi et al., 2016).

The precursor of M protein, prM, shields fusion peptide of E protein from acidic environment in trans-Golgi apparatus through its globular “pr” domain when newly budded virus particles transit through exocytosis pathway and thus prevents virus from fusing with Golgi apparatus prematurely. After that, the “pr” peptide is cleaved by a proprotein convertase, Furin, in Golgi apparatus and it dissociates from virus surface once particle reaches extracellular space (Gaucher et al., 2008; Yu et al., 2008, 2009). However, not only fully mature viral particles are produced during viral culture, partially mature or immature virions can also be detected in the supernatant. The immature viruses are those whose pr peptides were not fully proteolytic cleaved from all M proteins on surface. Functionally, in contrast to the mature virus, the immature DENV and ZIKV are non-infectious, however, such immature virus particle can also infect FcγR bearing cells after being bound by antibodies specific for “pr” peptides (Rodenhuis-Zybert et al., 2010; Wirawan et al., 2019), and the uptake of immature virion/antibody complexes creates mature, infectious viruses (Rodenhuis-Zybert et al., 2010).

Besides structural proteins incorporated into viral particles, another membrane associated glycoprotein NS1 can also be secreted extracellularly as hexamers, and detected in blood during acute infection. The NS1 protein can cause pathology in humans through either altering endothelial permeability or inducing antibodies that cross-react with host endothelial proteins; it can also facilitate viral transmission in mosquitoes through antagonizing mosquito innate immunity via suppressing the expression of immune-related genes involved in ROS and JAK/STAT pathways (Liu et al., 2011; Liu J. et al., 2016; Puerta-Guardo et al., 2019). Consistently, antibodies to NS1 can protect mice from lethal diseases in experimental models (Wan et al., 2017; Bailey et al., 2018).

## Cell Tropism of Dengue Virus and Zika Virus

Transmission of DENV and ZIKV are mainly mediated by *Aedes* mosquitoes. When human is bitten by infected mosquitoes, viruses are inoculated directly into the blood vessels or epidermis where keratinocytes, skin dendritic cells (DC) (Limon-Flores et al., 2005), monocytes and myeloid DC are probably infected and become the first virus carriers for further dissemination (Khaiboullina S. F. et al., 2019). An earlier investigation on dengue virus has indicated that immature skin DC, Langerhans cells were infected by dengue virus and then emigrated from the skin to transmit the virus to other organs (Wu et al., 2000). Our studies have demonstrated that monocyte was the principle target cell among PBMCs for both DENV and ZIKV infection, as well as the main mediators for ADE of both viruses in PBMCs (Kou et al., 2008; Li et al., 2018). Consistently, two other studies also provided evidences that circulating CD14 + monocytes were the primary cellular targets of ZIKV infection in PBMCs (Foo et al., 2017; Michlmayr et al., 2017).

As they disseminate through the circulatory and lymphatic system, dengue virus and Zika virus spread to different tissues and organs. Dengue virus infects primary vascular endothelial cells, splenic macrophage and Kupffer cells in *ex vivo* infection assay and immunohistochemistry of autopsy specimens (Marianneau et al., 1999; Jessie et al., 2004; Blackley et al., 2007; Dalrymple and Mackow, 2012). In contrast, besides these tissues, Zika virus also replicates in uterus and many immune privileged organs, including placenta, brain and testicles (Govero et al., 2016; Quicke et al., 2016; Sanchez-San Martin et al., 2018; Khaiboullina S. F. et al., 2019), causing neuropathology, congenital disorders, and damages to reproductive organs. So far, Zika virus has been found to infect a wide range of cell types, including neural progenitor cells, mature neurons and astrocytes in brain, Sertoli cells, Leydig cells, stem-like testicular peritubular cells, primary spermatocytes and spermatogonia in testicles, vaginal epithelium and uterine fibroblast in uterus, and trophoblasts, Hofbauer cells and endothelial cells in placenta (Ma et al., 2016; Miner and Diamond, 2017; Qian et al., 2017). Several investigations indicated Zika virus infects endothelial cells, the key component of blood tissue barriers, a fact that may help to explain how Zika virus crosses blood brain barrier and placental barrier (Liu S. et al., 2016; Papa et al., 2017). However, it is still controversial as to whether Zika virus infection alters endothelial barrier integrity. A recent study showed that ZIKV infection of human umbilical vein endothelial cells (HUVECs) increased endothelial permeability (Khaiboullina S. et al., 2019), whereas some other studies have demonstrated that Zika virus directionally infected polarized epithelial cells from apical side and egressed at basolateral side without a disruption of cell monolayer integrity (Tamhankar and Patterson, 2019).

Understanding of ZIKV cell tropism and dissemination across blood tissue barriers will help to better elucidate Zika pathology, and provide opportunity for the development of efficient treatment or prevention strategy. Meanwhile, pathogenic characteristics and tissue tropism should also been considered during development of prophylactic ZIKV vaccine. Its criteria for protective efficacies need to include not only reduced viral load and neuropathology, but also inhibited vertical transmission, and decreased damages to reproductive systems in animal models (Griffin et al., 2017; Shan et al., 2017; Zou et al., 2018). Different from DENV vaccine candidates, ZIKV vaccine might have to elicit higher level of immune responses (Shan et al., 2019) to overcome the special immune status (such as immune tolerance) of recipients during pregnancy (Nancy et al., 2012), and follow-up studies of the incidence of fetal microcephaly in women received ZIKV vaccine are also necessary in clinical trials.

## The Benefits and Limitations of the Only Licensed Dengue Vaccine

With effort that lasted two decades, the first licensed vaccine *Dengvaxia* (called CYD-TDV before licensure) has been developed by Sanofi Pasteur. It is a live attenuated vaccine comprised of four Dengue-Yellow Fever virus chimeras in one dose at 1:1:1:1 ratios, and it has been approved to use in over 20 dengue endemic countries and European Union and USA (World Health Organization [WHO], 2017; Halstead and Dans, 2019). Each component of this vaccine was developed on the backbone of Yellow Fever 17D vaccine by replacing prM and E genes with those from each serotype of dengue viruses (Guirakhoo et al., 2000; Guirakhoo et al., 2001). Through such a strategy, CYD-TDV was developed and showed to elicit neutralizing antibodies to four serotype of dengue viruses; moreover, this vaccines has high genetic stability, with limited mosquito transmission, and it is less hepatotropic (one major adverse effect of YF vaccine) (Guirakhoo et al., 2002; Brandler et al., 2005; Higgs et al., 2006; McGee et al., 2008; Mantel et al., 2011). In the large multicenter phase III trials, this vaccine had showed protective efficacies against symptomatic virologically confirmed dengue (VCD) of 54.8% in Asian, and 64.7% in Latin America; the general efficacies against hospitalization and severe dengue cases reached 80.3 and 95.5%, respectively, in Latin America (Capeding et al., 2014; Villar et al., 2015). Though not completely satisfactory, in light of the urgency and significant global demand of a dengue vaccine, CYD-TDV vaccine (*Dengvaxia*) was licensed at the end of 2015, accompanied by the WHO Strategic Advisory Group of Experts (SAGE) recommendation that this vaccine be used in all dengue-endemic counties (World Health Organization [WHO], 2017).

Despite filling in the void of dengue vaccine, its phase II and phase III trials revealed concerns regarding the vaccine’s safety. First, the immunogenicity and efficacy of this vaccine varied by age and prior dengue-infections: vaccinees of seronegative and children younger than 9 years’ old had increased risk of diseases (Hadinegoro et al., 2015). Vaccination of flavivirus seronegative individuals induced significant lower level of neutralizing antibodies comparing to those having pre-existing flavivirus immunity, specifically, the Geometric Mean Titer (GMTs) of seronegative groups were 47.4–90.8% lower than those of seropositive ones (Guirakhoo et al., 2006; Lanata et al., 2012; Dayan et al., 2013a; Hss et al., 2013). Notably, during long-term follow-up of participants of the two Phase III trials and one Phase IIb trial, vaccinated Asians between ages 2 and 5 years old have had an increased risk of hospitalization in the first year, with a relative risk of 7.5; and vaccinated Thai children between ages 4 and 5 had a relative risk of 2.44 in the 3rd year after vaccination. The vaccine efficacies in children younger than 9 years of age were significantly lower than those of 9 years or older according to a number of criteria (VCD:44.6% vs. 67.8%; hospitalization: 56.1% vs. 86.1%; DHF: 66.7% vs. 90.9%). Further analysis indicated that the lowest vaccine efficacy of 14.4% was in dengue seronegative children younger than 9 years of age (Hadinegoro et al., 2015). It is postulated that vaccination in dengue seronegative recipients had mimicked a course of asymptomatic primary dengue infection, which induced antibodies that enhance subsequent natural dengue infection. Consistently, after the large scale vaccination in Philippines, several vaccinated children and adults have died from lethal dengue diseases. By the time Sanofi Pasteur and WHO modified the instruction for *Dengvaxia* vaccination after those long-term follow-up studies, limiting its use to people who has a previous dengue infection (Vannice et al., 2018), 800,000 Philippine children had already been vaccinated, among them only 100,000 were seropositive, the rest were thus placed at the increased risk of severe DHF/DSS (Fatima and Syed, 2018). Second, *Dengvaxia* has a low protective efficacy against dengue-2 viruses that are endemic in Asia. In a Phase IIb trial, *Dengvaxia* showed poor protection against dengue-2 virus, reached an efficacy of merely 9%, despite having generated decent neutralization antibodies to DENV-2, with similar GMT (Geometric Mean Titer) as those specific for other serotypes when tested at 28 days after the final immunization (Sabchareon et al., 2012). Similarly, in a Phase III trial in Latin America, the GMTs of neutralization antibodies to DENV-1,-2,-3,-4 were 395, 574, 508, and 241, respectively, at 28 days after the 3rd injection; but the corresponding protective efficacy during the 1st year were 50.3, 42.3, 74, and 77.7% for each of the four serotypes (Villar et al., 2015). Based on these large scale clinical studies, an expected correlation between efficacies and neutralizing antibody titer PRNT (Plaque Reduction Neutralizing Test) values were not found for all four serotypes. It should be noted that a waning of cross-genotype neutralization was observed for monovalent CYD-2 vaccine induced antibodies at 6 months post immunization in a Phase I trial; specifically, GMT of neutralizing antibody specific for dengue-2 clinical isolates of heterologous genotype dropped as much as 10-fold compared with those that were specific for laboratory strain 16681 of autologous genotype (Guirakhoo et al., 2006). All these may indicate that high serotype-2 specific neutralization antibodies had been elicited by CYD-TDV, but it only sustained for a short time and was restricted to a limited number of genotypes. Such unsatisfied efficacy and lower neutralizing titers toward one serotype will also increase the risk of infection by that serotype, especially in those who are seronegative at the baseline. Third, although the highest level of protection was supposed to be achieved after the 3rd vaccination, its efficacy was found to be similar with that in peoples only received the 1st dose. How to modify the immunization schedule of this live attenuated vaccine to boost immune responses is still unclear, but it will probably be helpful to improve the efficacy of vaccine itself. Last but not the least, whether the emergence of Zika virus in DENV endemic regions and prior ZIKV immunity in vaccine recipients will affect the efficacy of *Dengvaxia* is unknown, and whether vaccination by *Dengvaxia* will enhance or prevent Zika virus infection is also uncertain. As co-circulation of two viruses and complicated interference between Dengue and Zika virus-specific immunity have already been observed (Bardina et al., 2017; Fowler et al., 2018; Li et al., 2018; Leborgne et al., 2019), it is worthwhile taking the probable influence of ZIKV into consideration during dengue vaccine development. Therefore, either the currently licensed dengue vaccine *Dengvaxia* or other new vaccines must be applied judiciously in epidemic regions.

## Other Dengue and Zika Vaccines Currently in Clinical Trials

### Other Dengue Vaccines in Clinical Trials

Besides the approved dengue vaccine *Dengvaxia*, several other dengue vaccine candidates have been advanced into clinical trials, these include two recombinant live attenuated vaccines, one subunit protein vaccine, and one DNA vaccine (Table 1).

**TABLE 1:** Dengue vaccines in clinical trials.

**Name**	**Vaccine type**	**Immunogen**	**Developer**	**Current status**
DENVax (TAK-003)	Live attenuated chimeric vaccine	Attenuated DENV-2; Substitution with DENV-1,3,4 prM and E genes on DENV-2 backbone;	Takeda Vaccine Inc.	Phase III
LAV Delta 30 (TV003/TV005)	Live attenuated chimeric vaccine	Attenuated DENV-1,3 and 4, through deletion in 3′UTR, DENV-2 prME on the backbone of attenuated DENV-4	NIAID/Butantan Institute	Phase III
DENV subunit V180	Subunit protein	Ectodomain (N-terminal 80%) of envelope protein, expressed in S2 cell line	Hawaii Biotech Inc./Merk	Phase I
D1ME100	DNA	prM and E genes	US Naval Research Center	Phase I

The live attenuated dengue vaccine TAK-003 (DENVax or TDV) is developed by Takeda Vaccine Incorporated. It is comprised of one attenuated dengue-2 viruses, and three other chimeras that contain prM and E genes of other three serotypes built on the backbone of attenuated DENV-2 (Osorio et al., 2015). Here, the backbone DENV-2 in TAK-003 is derived from dengue-2 attenuated virus strain PDK-53, which was obtained through serially passaging of DENV-2 16681 in primary dog kidney cells for 53 times (Bhamarapravati et al., 1987). This tetravalent vaccine has non-structural proteins of DENV-2, and it is capable of activating DENV-2 NS1 specific T cell responses and antibodies (Sharma et al., 2019), both of which *Dengvaxia* is incapable of inducing. But among tetravalent immune responses elicited by the chimeric TDV, the immunity to DENV-4 was the lowest when testing in Phase I and Phase II studies of tetravalent TDV formulations (Osorio et al., 2014; George et al., 2015; Sirivichayakul et al., 2016). Moreover, in the released data from part 1 of its ongoing phase III study, the efficacy of TAK-003 to DENV-4 was still unclear for the insufficient case numbers of DENV-4 infection during study (Biswal et al., 2019). Irrespective of this shortfall, the merits of this vaccine warrants its further testing in several Phase III trials in endemic regions.

A second tetravalent live attenuated dengue vaccine candidate is LAVDelta30 (TV003/TV005), invented by US NIAID and under co-development with Butantan Institute. This vaccine is comprised of attenuated DENV-1, DENV-3, and DENV-4 viruses that contain a 30-nucleotide deletion or additional 31-nucleotide deletion in 3′ UTR regions (rDEN1delta30, rDEN3delta30/31, rDEN4delta30) (Blaney et al., 2001, 2008; Whitehead et al., 2003a). Because the same deletion of 30 nucleotides in DENV-2 failed to attenuate it sufficiently, a chimeric virus was obtained by replacing prM and E genes of rDEN4delta30 with DENV-2 prM/E and used instead (Whitehead et al., 2003b). In completed trials and released results, this vaccine was reported to produce neutralizing antibodies and multi-type specific T cell responses in both seronegative and seropositive recipients, although GMTs of neutralizing antibody in seropositive recipients are always higher than those in seronegatives (Weiskopf et al., 2015a; Kirkpatrick et al., 2016). Generally safe, but the vaccine has induced adverse effects such as rash, neutropenia and short-time viremia (Durbin et al., 2005, 2006, 2011, 2013). Its first Phase III trial has been initiated in Brazil.

In contrast to the more rapid progress of live attenuated vaccines into Phase III clinical trials, protein and DNA vaccines are lagging behind in Phase I trials. A subunit dengue vaccine V180 is developed by Hawaii Biotech Inc., and then bought by Merk & Co., Incorporated. This vaccine is comprised of four *Drosophila* S2 cell expressed N-terminal 395aa of envelope proteins (E80) representing four serotype of dengue viruses, and it has been demonstrated to produce neutralizing antibody and provide protection against challenge with multiple serotypes of DENV in murine and non-human primate models (Clements et al., 2010; Govindarajan et al., 2015). In a recent Phase I clinical trial, tetravalent E80 vaccines formulated with different adjuvants were shown to be well tolerated in seronegative recipients in Australia, and they elicited tetravalent neutralizing antibodies, albeit of short duration (Manoff et al., 2019). Use this protein vaccine in adults who previously received attenuated vaccines, TV003 or TV005, has also been tested in clinical trial, the results of which are pending (ID: NCT02450838).

A DNA vaccine under clinical development is developed by US Naval Medical Research Center, and it contains prM and E genes. Monovalent DENV-1 prM and E DNA (D1ME) vaccines showed immunogenicity in Phase I clinical trial after being demonstrated conferring protection in a monkey model (Beckett et al., 2011). The neutralizing antibody was detectable only in the vaccine group receiving higher dose of DNA, and its titer was low. Therefore, tetravalent formulations of this vaccine were tested with combination of Vaxfectin^®^ adjuvant to improve its immunogenicity in further clinical trials (Porter et al., 2012), much of its results have not been published.

### Zika Vaccines in Clinical Trials

In contrast to Dengue vaccines, Zika vaccines have only been developed recently since the large ZIKV outbreaks in 2015–2016. Multiple candidates are under development simultaneously, including traditional inactivated vaccine, live attenuated vaccine, and genetically engineered subunit protein vaccine, DNA vaccine, and novel mRNA vaccines; among them, only the seven candidate vaccines that are in Phase I or II clinical trials will be discussed (Table 2).

**TABLE 2:** Zika vaccines in clinical trials.

**Name**	**Vaccine type**	**Immunogen**	**Developers**	**Current status**
VRC5283	DNA	Wild type prM and E genes, needle-free injection	NIAID	Phase II
GLS-5700	DNA	Consensus prM and E genes, injection & electroporation	GeneOne life science Inc./Inovio Pharmaceuticals	Phase I
ZIKV purified inactivated vaccine (ZPIV)	Inactivated vaccine	Formalin-inactivated whole virus	WRAIR/NIAID/Harvard University/Sanofi Pasteur	Phase I
VLA1601	Inactivated vaccine	Formalin-inactivated whole virus	Valneva Australia GmbH	Phase I
rZIKV/D4delta30	Chimeric live attenuated vaccine	ZIKV prM and E genes inserted to DEN4delta30	NIAID	Phase I
MV-ZIKA	Viral vector	ZIKV prM and E genges inserted to measles virus vector	Themis Bioscience GmbH	Phase I
mRNA-1325	mRNA	Lipid nanoparticle encapsulated modified mRNA of prM and E	ModernaTX, Inc.	Phase I

The most advanced Zika vaccine candidates is a DNA vaccine containing Zika virus prM and E genes (VRC5283) that is being developed by NIAID and tested in Phase II clinical trial. The vaccine plasmid expresses prM and E proteins and forms non-infectious but highly antigenic virus like particles (VLP) *in vivo*. Recipients of this vaccine by needle-free injection on both arms with split-dose showed 100% seroconversion, and generated neutralizing antibody with higher GMT values than those received a single-full dose injection on one arm and those administered the vaccine via needle and syringe injection. It is probably because the needle-free injection has enhanced dispersion field and increases the contact area between antigens and dermal APC (Mitragotri, 2006). The split-dose vaccination group also elicited best CD4 + and CD8 + T cell immune responses across all groups (Gaudinski et al., 2018). Another DNA vaccine GLS-5700 (developed by GeneOne/Inovio) containing consensus sequences of ZIKV prM and E genes, delivered via intradermal injection followed by electroporation, produced neutralizing antibodies in 62% participants as measured by the Plaque Reduction Neutralizing Test (PRNT). In addition, adoptive transfer of sera from vaccine recipients protected 92% immune deficient mice from lethal ZIKV challenge (Tebas et al., 2017).

The development of inactivated Zika vaccine was started soon after the ZIKV outbreaks. There are currently two purified inactivated Zika vaccine candidates in Phase I clinical trials, they were developed by WRAIR/NIAID/Harvard University/Sanofi Pasteur (ZPIV) and Valneva Australia GmbH (VLA1601) separately. Inactivated vaccine has the advantages of easy manufacture, established platforms based on many other vaccines, such as those for Japanese Encephalitis (JE) and Polio viruses, have been developed. According to preliminary results of the WRAIR/NIAID vaccine (ZPIV) in three Phase I trials, the formalin-inactivated virus vaccine induced 95% seroconversion, had a peak neutralization antibody GMT of 286, with only moderate adverse effect. Adoptive transfer of human IgG from vaccine recipients to mice prior to challenge with ZIKV led to reduced viremia in mice, indicating its potential for protection in humans (Modjarrad et al., 2018).

Using a similar strategy for DENV-2 vaccine TV003/TV005, recombinant live attenuated Zika vaccine has been constructed with the backbone of attenuated DENV-4 of TV003 (rZIKV/D4delta30), by NIAID, wherein prM and E genes of DEN4delta30 were substituted by the corresponding genes from ZIKV. This vaccine is now in Phase I clinical trial, and might be tested in combination with DENV vaccine TV005 in the future (ID: NCT03611946) (Durbin and Wilder-Smith, 2017; Wilder-Smith et al., 2018). Other viral vectors such as Measles vaccine virus has also been adapted to construct chimeric ZIKV vaccine. One of such vaccine, MV-ZIKA, has been developed by Themis Bioscience GmbH and advanced into Phase I clinical trial (ID: NCT02996890).

Novel vaccine strategy such as lipid nanoparticle-encapsulated modified mRNA has also been used to develop Zika vaccine. An mRNA that contains prM-E genes of ZIKV and optimized 5′ and 3′ untranslated sequences with type-1 cap was obtained through enzymatically synthesis using modified nucleoside, and packaged into lipid nanoparticles (LNP). Such mRNA-LNP vaccine could efficiently produce virus like particle in cells. After intramuscular delivery, two doses of mRNA-LNP vaccine were able to elicit high titers (∼1/100,000) of neutralizing antibodies, and conferred protection against ZIKV challenge in mice (Richner et al., 2017a). An mRNA-based vaccine is now in Phase I clinical trial (ID: NCT03014089).

Besides, other forms of ZIKV vaccine, such as protein based subunit vaccine (Medina et al., 2018; Metz et al., 2019; Slon-Campos et al., 2019) and live attenuated Zika vaccines (Richner et al., 2017b; Xie et al., 2018; Shan et al., 2019) are also under development at the pre-clinical stage. Considering the cross-reactivity and ADE between ZIKV and DENV, newly developed ZIKV vaccine should also be tested for DENV enhancement activity. This additional requirement will likely to be a huge obstacle for both ZIKV and DENV vaccines.

## Protective Immunity Against Dengue and Zika Viruses

### Protective Antibody Response

Although various antibodies targeting different viral proteins are induced after flavivirus infection, only two types of antibodies have been documented to provide significant protection: neutralizing antibodies to viral surface E protein (Crill and Chang, 2004; Dejnirattisai et al., 2015; Stettler et al., 2016; Robbiani et al., 2017) and non-neutralizing antibodies specific for NS1 protein (Henchal et al., 1988; Beatty et al., 2015; Wan et al., 2017; Bailey et al., 2018).

The ectodomain of flavivirus envelope protein can be structurally divided into three sub-domains: Domain II is a long finger-like area lying parallel to virus surface and it contains a fusion peptide on its tip; Domain III is an immunoglobulin-like folded structure projecting slightly away from virus surface, and it is linked to the C-terminal stem region and thought to be involved in virus binding; Domain I is an eight-stranded β-barrel that connects domain II and domain III. Previous study with dengue specific mouse monoclonal antibodies indicated that E domain III was the predominant targets for DENV neutralizing antibodies, and antibodies to this region are mainly serotype specific (Lok et al., 2008); whereas antibodies specific for fusion loop epitopes (FLE) are usually highly cross-reactive with modest neutralization activities (Crill and Chang, 2004). However, in convalescent dengue patient, anti-E Domain III specific neutralizing antibodies only constitute a small proportion of DENV antibody repertoire. Mapping antibody epitope using recombinant envelope proteins, it was found that neutralizing antibody epitopes in human distributed widely across both domain III and domain I/II regions (Beltramello et al., 2010; Stettler et al., 2016), and the depletion of EDIII specific antibodies did not affect the neutralization activity of human immune sera significantly (Williams et al., 2012). Subsequent studies with human samples revealed another class of antibodies that target quaternary epitopes on virus surface, and that these antibodies do not bind to recombinant envelope proteins, but recognize epitopes spanning one dimer or two adjacent dimers of E proteins on the intact virus surface. One group of such antibodies, also called envelope dimer epitope (EDE) antibodies, usually have broad neutralizing activities to all four serotype dengue viruses (Dejnirattisai et al., 2015; Rouvinski et al., 2015).

On the contrary, the importance of ZIKV EDIII specific antibodies has been shown directly in some human studies. In one study with a panel of human mAb isolated from four convalescent patients, antibodies to E domain III and quaternary epitopes were found to be the most potent at neutralization (Stettler et al., 2016). Additionally, using two cohorts of ZIKV infected individuals from Mexico and Brazil, it was revealed that the levels of antibodies specific for the lateral ridge of ZIKV E domain III were positively correlated with sera neutralization antibody titers (Robbiani et al., 2017). Moreover, antibodies to quaternary epitopes formed between E domains II and III of Zika virus were demonstrated to be both protective and therapeutic in murine models (Sapparapu et al., 2016; Hasan et al., 2017; Collins et al., 2019).

**FIGURE 1:**
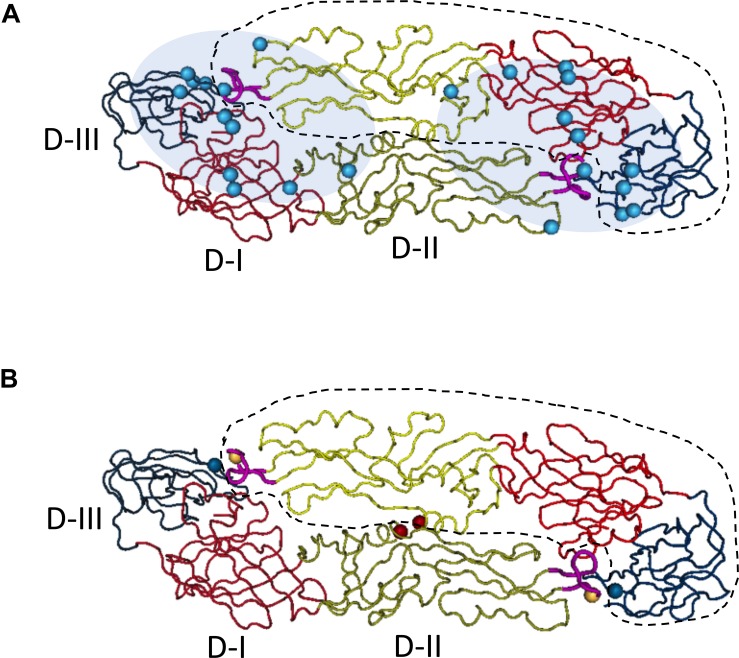
Quaternary epitopes on dengue virus dimeric envelope proteins and stabilizing dimeric structure of E proteins through the addition of disulfate bonds between two monomers. Based on the published structure of DENV-2 E dimer (PDB access NO:1OAN), three domains of E monomer were denoted with different colors on backbones (D-I, red; D-II, yellow; D-III, blue). One of the E monomers in the dimeric structure was circled with dash line. On the tip of domain II, the fusion loop was labeled in pink. The structural image presented the top view, distal from virus membrane. **(A)** Epitopes distribution of EDE epitopes, the residues (cyan spheres) critical for EDE antibody binding were determined by Dejnirattisai et al. Fab binding regions of EDE antibodies were mapped to either end of the dimer (cyan shadow), covering the interface of two monomers. Figure adapted from Dejnirattisai et al. (2015) and Rouvinski et al. (2015). **(B)** Stabilization of dimeric E proteins was achieved through adding disulfate bonds, after introducing mutations into domain II, L107C (yellow spheres) A259C (red spheres) and domain III, A313C (blue spheres). Figure adapted from Rouvinski et al. (2017).

DENV and ZIKV show high level of structure homology between their envelope proteins, and share 35, 51, and 29% amino acid identity in EDI, EDII, and EDIII, respectively. Thereby, cross-reactivities are frequently observed between antibodies targeting E domain I/II of two viruses, but less so for domain III antibodies (Stettler et al., 2016). The broadly neutralizing EDE1 antibodies, one subset of EDE antibodies originally isolated from DENV patients, has been shown to potently neutralize Zika virus *in vitro* and provides protection against lethal challenge of Zika virus in mice. These antibodies recognize a conserved conformational region on the E dimer which prM interacts with during virus maturation. Another subset is EDE2 antibodies that have broader footprints covering N153 glycosylation site of DENV E proteins, these antibodies can also cross-neutralize ZIKV at high concentrations (Fernandez et al., 2017; Abbink et al., 2018; Figure 1A). In contrast, ZIKV neutralization antibodies targeting quaternary epitopes identified so far, such as ZIKV-117, A9E, and G9E, are mainly type-specific (Sapparapu et al., 2016; Hasan et al., 2017; Collins et al., 2019). Notably, cross-reactive antibodies EDE1 and EDE2 were isolated mainly from patients of acute secondary DENV infection, whereas A9E and G9E were isolated from DENV naïve ZIKV patients at 6 months after the onset of illness. Whether such cross-complex neutralizing B cell responses can sustain long time after recovery from infection is unclear, and whether its frequency increases after a heterotypic secondary infection is also unknown.

Collectively, neutralization antibodies to dengue or Zika viruses are mainly constituted of type specific EDIII antibodies, cross-reactive EDI/II antibodies, and potently neutralizing quaternary epitope antibodies that often neutralize four serotypes of dengue viruses simultaneously.

Other than antibodies to envelope proteins, there are abundant prM specific antibodies in human sera after dengue virus infection (Dejnirattisai et al., 2010). However, such antibodies are poorly neutralizing with high cross-reactivity, and induces ADE in a wide range of concentrations, probably because prM antibodies facilitate non-infectious immature virus to become mature and infectious (Rodenhuis-Zybert et al., 2010). In experimental models, immature viral particles may achieve infectivity with the help of antibody against “pr” upon entering the acidic endosome, where the “pr” peptide is cleaved together with the antibody by Furin. Even partially mature viruses that keeping pr peptides in some region on viral surface, can also obtain enhanced infection in Fcγ receptor bearing cells with the help of anti-pr antibodies (Rodenhuis-Zybert et al., 2010; Wirawan et al., 2019). Because of these reasons, prM antibody is now thought to be undesirable among vaccine induced immune responses.

### Antibody Dependent Enhancement

One adverse characteristic of flavivirus prM or E antibodies is their ability to induce ADE. When the concentration or affinity of antibodies is too low to neutralize virus infection, the immune complexes formed by virus and antibodies tend to interact with Fcγ receptors on myeloid cell surface through Fc domains of antibodies, they do not induce Fcγ-mediated viral clearance, but aid virus infection by directly increasing virus uptake through Fcγ receptor, or boosting virus replication intracellularly via activating downstream pathway to antagonize the innate immunity (Halstead, 1988; Kliks, 1990). In the latter case, it is hypothesized that legation of Fcγ receptor transmits signals through spleen tyrosine kinase (SYK) to activate extracellular signal-regulated kinase (ERK), and subsequently enhances the transcription of genes including IL-10, which antagonizes type-I IFN pathway through stimulating members of the suppressor of cytokine signaling (SOCs) (Halstead et al., 2010). A number of *in vitro* studies have already demonstrated that most antibodies to DENV or ZIKV prM and E proteins have the potential to enhance viral infections at sub-neutralizing concentrations, irrespective of their neutralization potency (Pierson et al., 2007; Dejnirattisai et al., 2015, 2016). Two classes of antibodies are thought to induce mainly ADE effect rather than neutralization, these are antibodies to fusion-loop and antibodies specific for “pr” peptide, both of which are abundant in patient sera (Beltramello et al., 2010; Dejnirattisai et al., 2010). They are generally highly cross-reactive and marginally neutralizing (Rodenhuis-Zybert et al., 2010; de Alwis et al., 2014). Further investigation on Zika and dengue monoclonal antibodies indicates that antibody concentrations that induce peak ADE are usually negatively correlated with antibodies’ neutralizing capacity (Li et al., 2017).

In addition to *in vitro* studies using human samples, ADE of heterotypic DENV infection has been more stringently demonstrated in mouse models. Administering heterotypic DENV immune sera or viral-specific monoclonal antibodies to AG129 immune deficient mice prior to infection with non-lethal dose of DENV-2 increased viral replication and enhanced disease severity; and increased infection of liver sinusoidal endothelial cells were observed for all four serotypes of clinical isolates (Zellweger et al., 2010). Consistently, clinical evidence of ADE in DENV infection has been reported previously. Infants born to dengue immune mother had elevated risk of DHF/DSS at 6–9 months of age, due to the decay of maternal-derived DENV antibodies (Halstead, 2003). Further, the increased risk of hospitalization in younger children vaccinated with CYD-TDV in Phase III clinical trials are also suspected to have been caused by ADE, as a result of vaccine-induced poorly neutralizing antibodies. More recently, a long-term study on a Nicaragua pediatric cohort showed that the risk of severe dengue diseases was highest within a narrow range of preexisting anti-DENV antibody titers, affirming antibody concentration is a critical determinant of ADE (Katzelnick et al., 2017).

More significantly, a cross-serocomplex ADE between ZIKV and DENV has also been reproduced in mouse model. DENV and WNV immune sera were both found to enhance Zika virus infection in mice (Bardina et al., 2017). Also, the presence of DENV-specific antibodies in ZIKV-infected pregnant mice significantly enhanced viral replication in placenta, and increased placenta damage, reduced fetal growth, and accelerated fetal resorption (Leborgne et al., 2019). Reciprocally, maternally acquired Zika virus antibodies also enhanced dengue disease severity in mice (Fowler et al., 2018). In non-human primate models, the results are not as clear. Two studies did not show enhanced Zika virus infection in macaques that were infected by DENV either 420 days or 2.8 years before experiments (McCracken et al., 2017; Pantoja et al., 2017). However, it should be noted that the interval between primary and secondary DENV infections is critical for disease severity, and DENV ADE in human has only occurred within a relatively narrow range of pre-existing DENV antibody concentration (Katzelnick et al., 2017). In dengue infected patients, this interval was usually 12 months or longer, but unknown for enhancement of Zika virus infection. Limited data from a long-term cohort study in Brazil before and after the 2015 ZIKV outbreak have found that the pre-existing DENV NS1 specific IgG3 antibody within 4–6 months of infection was positively associated with the risk of subsequent ZIKV infection, but high titer of total IgG to DENV was associated with protection against the acquisition of ZIKV infection (Rodriguez-Barraquer et al., 2019). Our previous study using sera of convalescent dengue patients also revealed that stronger cross-serocomplex ADE of ZIKV infection in PBMC appeared more often in sera obtained within 3 months of DENV infection (Li et al., 2018). These results are supportive of the idea that concentrations of cross-reactive antibodies or intervals between two infections are important factors for determining cross-serocomplex enhancement between ZIKV and DENV.

Collectively, ADE is now considered to be the underlying mechanism for enhanced risk of DHF/DSS in secondary dengue virus infection, and it highlights the necessity of avoiding the induction of inadequate immune response toward any serotypes during vaccine development. Despite the impact of prior cross-serocomplex immunity for subsequent ZIKV or DENV infections are clinically unknown, candidate vaccines that provide protection and avoid ADE should be prioritized for development.

### T Cell Responses

Aside from the double-edged antibody responses, DENV and ZIKV infections also elicit strong T cell responses of dubious functions. For a long time, T cells activated during dengue virus infection was postulated to cause pathology through producing cytokines such as IFN-γ and TNF-α, which in some models directly increases vascular permeability (Rothman et al., 2014). However, in clinical studies, the appearance of T cell responses was found after the occurrence of hemoconcentration or thrombocytopenia in DHF patients (Jin, 2008; Dung et al., 2010). In mouse models, DENV-specific CD8 + T cell responses were protective and essential for virus clearance during primary infection; and CD4 + T cell responses elicited after peptide vaccination also contributed to protection, although they seemed to be dispensable for virus clearance in primary infection (Yauch et al., 2009; Yauch et al., 2010). A subsequent human cohort study further illustrated that DENV specific CD8 + T cell responses played protective roles against DENV infection in an HLA-linked manner (Weiskopf et al., 2013). DENV-1, -2, -4 specific human CD8 + T cell epitopes are distributed predominantly on NS3, NS4B and NS5 proteins (Duangchinda et al., 2010; Rivino et al., 2013; Weiskopf et al., 2013), whereas human CD4 + T cell epitopes are mainly located on C, NS3, and NS5 viral proteins (Weiskopf et al., 2016; Grifoni et al., 2017a). In some studies, DENV-3 specific CD8 + T cell responses were found to target both structural and non-structural proteins (Weiskopf et al., 2014, 2015b). Of note, all these observations were based on limited sample size and HLA types, and of unknown sequences of heterotypic infections when the subjects had experience secondary infections. These uncertainty in clinical studies might have not revealed the full scope of T cell responses toward conserved epitopes.

Similarly, ZIKV specific CD8 + T cells were also first showed to be protective in a mouse model (Elong Ngono et al., 2017; Huang et al., 2017), and human ZIKV CD8 + T cells were revealed to have cytotoxic antiviral function and bear signature transcriptional profiles (Grifoni et al., 2018). Though, CD8 + T cell infiltration into mouse brain and its antiviral cytotoxicity might be associated with neurological pathology in ZIKV infection, the pre-existence of specific CD8 + T cells did protect mice from CNS disease (Huang et al., 2017; Jurado et al., 2018). Whether ZIKV CD4 + T cell responses are necessary for virus clearance in primary infection is still controversial, but memory CD4 + T cells elicited by infection or peptide immunization have been demonstrated to protect host from subsequent ZIKV infection (Hassert et al., 2018; Elong Ngono et al., 2019). In one cohort study conducted in dengue endemic countries, ZIKV specific CD8 + T cell epitopes were found to be located mainly on C, prM, and E proteins in dengue naïve patients, but targeted broadly across the proteome, especially those conserved regions, in dengue experienced patients (Grifoni et al., 2017b). This is in accord with observations made in human HLA-transgenic mice (Wen et al., 2017b).

Cross-reactive T cell responses were often observed between DENV and ZIKV, because of their homology at amino acid level. DENV specific memory CD8 + T cells could cross-react with Zika virus and protected mice from subsequent lethal ZIKV challenge (Wen et al., 2017a). Adoptive transfer of DENV CD8 + T cells to pregnant mice inhibited Zika virus replication in placenta and increased the survival of fetus (Regla-Nava et al., 2018). In DENV convalescent patients, memory CD4 + and CD8 + T cells could be activated by peptides derived from ZIKV capsid and NS3 proteins, and the activated CD8 + T cells could kill ZIKV infected cells *in vitro* (Lim et al., 2018). Nevertheless, studies on the cross-reactive CD4 + T cells are few, evidence for ZIKV specific memory T cells cross-react with DENV antigens is still lacking.

All above demonstrate that T cell response is an important element of adaptive immunity toward dengue and Zika virus, and T cell component should be considered in the development of protective vaccines.

## B and T Cell Vaccines of the Next-Generation

Based on the existing knowledge of vaccine immunology and protective immunity to DENV and ZIKV, it is predicted that an ideal dengue or Zika vaccine should induce both humoral and cellular immune response to ensure full protection.

### Universal B Cell Vaccines for DENV and ZIKV

Previous investigation on dengue and Zika vaccine mainly focused on the B cell immunity, aiming to elicit enough neutralizing antibodies for *in vivo* protection. Because of the complex interaction among antibody responses to four DENV serotypes and ZIKV, an ideal dengue vaccine should elicit long-lasting, serotype-specific neutralizing antibodies to each serotype of virus or broadly neutralizing antibodies that cross-react with all serotypes of viruses. Zika vaccines developed independently need to be able to trigger autologous neutralizing antibodies but minimal cross-reactivity with dengue viruses. Alternatively, pentavalent vaccines would be necessary, if ADE cross sero-complex is verified in epidemiological studies in the future. However, to induce long-term B cell memory response that sustains potent neutralization is not easy, and to produce adequate and relatively balanced neutralizing antibodies against each of these viruses is also difficult. The following approaches may be considered.

First, epitope selection is necessary to design vaccines that produce strong neutralizing antibody responses and minimal ADE effect. Traditional live attenuated vaccines have the advantages of strong immunogenicity and mimicry of wildtype virus. However, the antibody responses elicited by intact or recombinant dengue or Zika viruses still maintain the dual functions of neutralization and enhancement. One prominent source of antigens that induce enhancing antibodies is the immature or partially mature viral particles within the vaccine produced *in vitro* or viruses replicated *in vivo*, and the percentages of which are difficult to determine due to variability in cell cultures for different virus strains. Epitopes on “pr” peptides are probably exposed on immature ZIKV virus surface and elicit abundant ADE inducing antibodies, similar to what happened in DENV infected patients. The same issue must be dealt with for DNA or RNA vaccines that incorporate prM and E genes in order to make virus like particles *in vivo* (Beckett et al., 2011; Porter et al., 2012; Richner et al., 2017a). Similarly, *in vitro* produced virus like particle vaccines will not be ideal unless the immature virus-like particle can be eliminated through procedures such as exogenous Furin cleavage, but how to produce enough immunogens is another problem even more complicated to deal with (Urakami et al., 2017). Therefore, the exact effect and ratio of “pr” antibodies elicited by these vaccines have to be determined during development. In comparison, subunit proteins are easier for epitope selection and quality control. In most subunit candidate vaccines of DENV and ZIKV, soluble EDIII or E80 as antigens were chosen (Block et al., 2010; Liang et al., 2018; Manoff et al., 2019) to induce neutralizing antibodies that block virus attachment or/and inhibit post-entry membrane fusion. Nevertheless, fusion-loop epitope antibodies can still be induced by E80 due to the probable re-exposure of fusion-loop under acidic environment even for an envelope dimer immunogen. One study has attempted to abolish fusion loop through site-directed mutation in mRNA vaccine (Richner et al., 2017a), but both neutralization and enhancement activities were attenuated, indicating this region was probably essential for E dimeric structure on VLP surface. Alternatively, cross-linking of two E80 proteins through additional disulfate bonds in domain II and domain III could stabilize the anti-parallel structure of the soluble E80 dimer, limiting the exposure of fusion-loop, but enabling the presentation of cross-serocomplex broadly neutralizing EDE epitopes (Rouvinski et al., 2017; Figure 1B). Consistently, in a recent study, the covalently stabilized ZIKV E dimer has successfully elicited protective antibody responses against ZIKV infection in mice, without causing cross-reactivity to dengue viruses or ADE of DENV (Slon-Campos et al., 2019). Currently, both Zika EDIII and Dengue tetravalent EDIII vaccines are under development. The EDIII component theoretically has more serotype/complex specificity and produce less ADE effect. Whether these type-specific neutralization antibodies induced are strong enough to provide *in vivo* protection toward multiple viruses is to be determined.

Second, relatively balanced neutralization against each of the viruses is required for an ideal tetravalent dengue vaccine or pentavalent Dengue/Zika vaccine. The majority of dengue vaccine candidates are comprised of mixed antigens representing four serotypes, and they are administered to recipients simultaneously to elicit a tetravalent response. When using attenuated viruses, however, different replicative capacity of each virus may produce interference among them, leading to imbalanced neutralizing antibody responses (Dayan et al., 2013b). As for tetravalent protein or DNA vaccines, the immune dominance of specific antigens also affected the balance of neutralizing activities elicited by the vaccines (Block et al., 2010). Thus, modulation of dosage of each serotype of virus or antigen is always needed to improve the relative balance of antibody responses induced. Because the correlation between neutralizing titers and *in vivo* protection varies among four serotypes of dengue viruses or Zika virus, to adjust the balance among immune responses in order to achieve broad protection becomes even more difficult. An alternative strategy is to elicit broadly neutralizing antibodies through a single antigen presenting conserved neutralizing epitopes. To this end, we obtained a consensus E80 sequence that contains epitopes conserved across all four serotype viruses through *in silicon* calculation with a dataset composed of 3,127 published sequences of dengue viruses, and found this single consensus E80 protein was immunogenic and capable of inducing neutralizing antibodies toward all four serotypes of DENV, with less bias than conventional tetravalent E80 vaccine (Sun et al., 2017). When administered in a DNA prime and protein boost regimen, this vaccine conferred protections against all four serotypes of viruses in a mouse model (Wang et al., 2019). This vaccine design strategy uniformly enriched all conserved epitopes on a single E80, further incorporation of consensus E80 monomers derived from both DENV and ZIKV to a stabilized dimer might help to present most cross-neutralizing epitopes on surface.

Moreover, to elicit long-term neutralizing B cell memory response is another essential aim for dengue or Zika vaccine. In clinical trial of CYD-TDV, GMT of antibody to each serotype of viruses was measured at 28 days after each immunization, significant increase of GMT level was only observed after the 1st and 2nd immunization, and protection efficacy in recipients accepted all three doses (per protocol analysis) showed no differences with those only vaccinated for the first dose (intention-to-treat analysis) (Capeding et al., 2014; Villar et al., 2015). Whether B cell memory response elicited by multiple and single doses of CYD-TDV both waned to the same level in the first year is uncertain. For obvious reasons, dengue experienced vaccine recipients have higher GMT than dengue naïve ones, and the formers were better protected from subsequent dengue infections. In an earlier Phase I clinical trial with monovalent CYD-2 vaccine, dramatic decrease of neutralizing antibodies was observed in YF naïve group from 1st to 12th month, whereas YF-immune subjects maintained higher GMTs of antibody responses to dengue virus-2 and heterotypic viruses throughout the first year after immunization, despite the vaccine specific GMT were initially similar for the two groups in the 1st month (Guirakhoo et al., 2006). More followed-up studies are needed for confirmation, but it inferred that boosting with heterologous antigens (i.e., vaccinate recipients having prior flavivirus infection) have helped host immunity to focus on a few conserved epitopes to produce stronger and longer cross-reactive broadly neutralization responses, but repetition of immunization with same antigens (i.e., various antibody responses primed in the first immunization were equally boosted in the following two repeated immunization) only elicited various antibodies of weak cross-neutralization and sustained these responses only for a short-term. In fact, this hypothesis is in line with the theory of “original antigenic sin” (Halstead et al., 1983; Kuno et al., 1993; Zompi and Harris, 2013). Therefore, to achieve long-term antibody responses by B cells toward selected epitopes corresponding to stronger and broader neutralization, heterologous prime-boost immunization to highlight conserved epitopes might be a useful approach.

In summary, through enriching conserved epitopes and selectively presenting broadly neutralizing epitopes, we should be able to construct a universal dengue and Zika B cell vaccine. Furthermore, with suitable heterologous vaccines in prime-and-boost regimens, we might have a chance to achieve the induction of potent, broadly reactive, and protective B cell immune responses that have limited ADE effect.

### T Cell Vaccines for DENV and ZIKV

In contrast to the extensive attention on B cell responses, T cell immunity produced by flavivirus vaccine had never been carefully examined until a recent retrospective study that investigated why there was a higher efficacy of CYD-TDV in dengue experienced than dengue naïve vaccine recipients. As a chimera of Dengue virus and YF-17D vaccine, CYD-TDV mainly elicited YFV specific T cell responses through YFV non-structural proteins, of which reactivity to dengue virus was limited. One hypothesis is that, with prior dengue virus infection, dengue specific memory T cell responses were boosted by YFV cross-reactive epitopes in seropositive recipients and contributed to protection. Indeed, the cross-serotype and cross-complex protection of dengue virus specific T cells has already been reported in mouse models. Specifically, memory T cells can relieve mice from lethal disease in an adoptive transfer model, avoiding ADE effect successfully (Wen et al., 2017a). Therefore, T cell vaccine is complementary to traditional B cell vaccines for defense against DENV and ZIKV.

**FIGURE 2:**
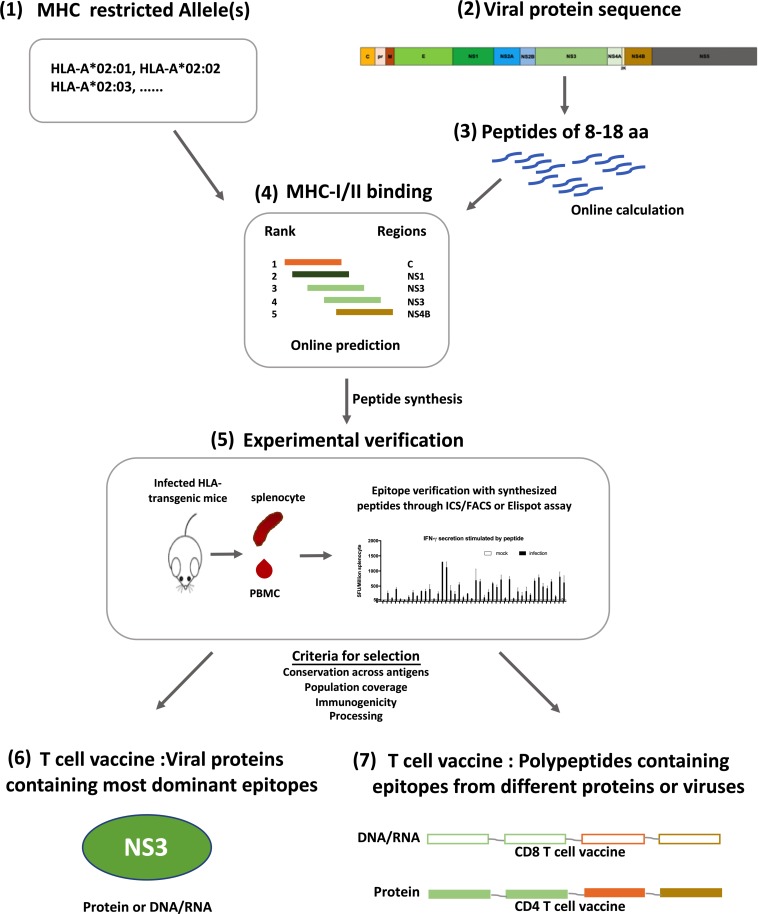
Designing strategy for flavivirus T cell vaccines. Development of T cell vaccine could be initiated from the prediction of T cell epitopes on Immune Epitope Database (IEDB) (1–4). With selected HLA molecules (1) and protein sequences (2), both CD4 + and CD8 + T cell epitopes can be predicted according to the sequences of linear peptides derived from a protein (3), and the affinity of peptides to selected MHC molecules (4). Predicted epitopes were verified in HLA-transgenic mice with synthesized peptides through Elispot or Intracellular cytokine staining assay (5). Verified epitopes were further selected based on their conservation across different strains of viruses (HLA-allotype), population coverage, immunogenicity and processing efficiency, all of which could be calculated online. Some can be tested experimentally. Using these selected epitopes, T cell vaccine can be prepared with two different strategies, one is using antigens which contain most dominant epitopes and have reliable population coverage and sequence conservation (6); the other is to make immunogens with a string of peptides containing epitopes from different genes or strains, thus having higher population and antigen coverage (7). Both kinds of vaccines can be either DNA/RNA or proteins, depending on the T cell subset to be activated.

Distinct from B cell epitopes, T cell epitopes are linear, continuous and major histocompatibility complex (MHC) antigen (human leukocyte antigen, HLA, in humans) restricted. Some HLA-linked protective epitopes have already been identified for DENV and ZIKV, it is worthwhile to study epitope distribution for more HLA allotypes. This can be initiated through predictive computational algorithms according to parameters such as MHC affinity, and followed by experimental verification (Elong Ngono et al., 2017, 2019). Viral proteins that contain most epitopes for common HLA allotypes could be included as antigens for T cell vaccines (Figure 2). Such an approach had been tested in a study of immunogenicity and protection efficacy of multiepitope DENV NS3 DNA vaccine in Balb/c mice, and it demonstrated that *in vivo* expression of NS3 epitopes elicited T cell responses and protective immunity against DENV-2 infection (Costa et al., 2011). Dominant epitopes from different proteins of different viruses presented by various HLA allotypes can also be integrated into a polypeptide string, which can be made into immunogens to elicit protective T cell immunity in a large population, just as what has been done for HIV CD4 + T cell vaccine (Liu H. et al., 2017).

Live attenuated vaccine has an advantage on eliciting T cell responses. TAK-003 and TV003 containing non-structural proteins of DENV have been shown to elicit both antibody responses and T cell responses. However, to avoid ADE, a flavivirus T cell vaccine without cell surface epitopes is obviously more suitable for the proof-of-concept study. To accomplish this goal, many factors affecting the immunogenicity of T cell antigens should be considered. First, immunogens activating two main subsets of T cells have to be delivered in different ways. CD4 + T cell epitopes are mainly presented through an exogenous antigen presentation pathway, whereas CD8 + T cell epitopes are presented more efficiently through an endogenous antigen presentation pathway (Cresswell et al., 2005; Roche and Furuta, 2015; Sadegh-Nasseri and Kim, 2019). Therefore, CD4 + T cell antigens should be administered either directly to extracellular space or secreted to extracellular space after *in vivo* expression. On the other hand, CD8 + T cell antigens are best delivered as DNA or RNA vaccines that have better antigen expression and retention intracellularly. Second, immune dominance of T cell epitopes might be affected by epitope composition of vaccines, and will also be influenced by prior flavivirus infection. How to induce protective T cell immunity toward four serotypes of DENV and ZIKV simultaneously, and whether cross-reactive T cell responses could be induced through heterologous prime-boost vaccination strategy, are all questions that have to be addressed during flavivirus T cell vaccine development. Finally, to achieve sterilizing immunity against viruses is extremely hard through T cell vaccine alone, as the effector T cells are only activated by processed immunogens and only target infected cells bearing linear epitopes on surface, both of which happen after cells were infected.

In summary, we propose an improvement of the strategy for dengue and Zika virus vaccine development by inclusion of T cell epitopes in order to overcome the ADE effect *in vivo*.

## Vector Blockade Through NS1 Antibodies

As both DENV and ZIKV are transmitted through *Aedes* mosquitoes, countermeasures that block the transmission steps of these vectors are important adjuncts to vaccines or antiviral drugs for controlling their infections. In urban areas, through spraying insecticides and clearing up standing water in containers such as discarded tires and plant pots, adult mosquitoes and mosquito larvae can be effectively removed. These approaches had helped to eliminate dengue fever from southern United States in 1920s. However, absolute elimination of mosquitoes from cities was impossible, reintroduction occurred when global travel became frequent and the new vector, *A. albopictus*, was imported from Asia. Nowadays, investigators are trying to sterilize mosquitoes through genetically engineered endosymbiotic bacteria *Wolbachia* (Zheng et al., 2019), but debate on its long-term ecological influence is continuing.

Leaving the eco-system intact, our investigations on NS1 provided an alternative strategy to block flavivirus-mosquito transmission which is more specific and environmentally friendly. Soluble NS1 hexamers are secreted during virus infection, and a high concentration of plasma NS1 coexists with viremia during the acute phase of disease. We demonstrated that blood meal from infected mice that have NS1 antigenemia can suppress the expression of immune genes (JAK-STAT, ROS) in midgut of mosquitoes, facilitating flavivirus acquisition by mosquitoes. Although transferring NS1 antibody to AG6 mice did not affect the viremia in mice directly, it significantly inhibited DENV-2 transmission from AG6 mice to mosquitoes (Liu J. et al., 2016). Immunization generated NS1 antibodies might competitively bind to soluble NS1 in blood and prevent NS1 from suppressing the expression of immune genes in midgut of mosquitoes after a blood meal. Furthermore, we demonstrated that NS1 antigenemia also determined ZIKV infectivity in mosquitoes. Specifically, comparing with ZIKV Asian strains that were isolated before recent outbreaks, contemporary strains causing recent epidemics have an alanine-to-valine substitution at the 188th residue in NS1. This mutation results in higher level of NS1 secretion, which subsequently dampens the expression of immune genes in mosquito and is responsible for the enhanced infectivity in mosquitoes. This mechanism helps to explain how ZIKV of Asian lineage acquired the ability to rapidly spread from Asia to America (Liu Y. et al., 2017).

Besides blockade of viral transmission, NS1 antibodies have been identified to have multiple other roles in immunity and host pathology, some of which are completely opposite. Previously, soluble DENV NS1 was found to contribute directly to vascular leakage through increasing endothelial permeability and degradation of endothelial glycocalyx components, or indirectly through inducing vasoactive cytokines (TNF-α, IL-1β, and IL-6) via TLR-4 activation (Beatty et al., 2015; Modhiran et al., 2015; Glasner et al., 2017). Further study on DENV, WNV, YFV, JEV, and ZIKV revealed that the effect of NS1 on cell permeability was probably universal in flavivirus but had tissue-specificity and thus there are different disease patterns for different viruses (Puerta-Guardo et al., 2019). In addition, DENV NS1 also facilitates viral immune evasion through inhibiting complement cascade in mammals (Avirutnan et al., 2010). Therefore, blocking NS1 through specific antibodies has been shown to attenuate plasma leakage and mortality in dengue infected mice (Henchal et al., 1988; Wan et al., 2017). Because a fraction of NS1 dimer also presents on the surface of infected cells, antibodies also probably target Fcγ-receptors to mediate viral clearance by phagocytosis or initiate complement dependent cytotoxicity (CDC). Recent investigations show that transferring ZIKV NS1 antibodies from human or vaccinated mice to IFN deficient mice provided protection through Fcγ-receptor mediated pathway (Bailey et al., 2018, 2019). On the contrary, much evidence also indicated NS1 antibodies being capable of cross-react with coagulation factors and adhesin molecules on platelets and endothelial cells, causing disruption of platelet aggregation and apoptosis of endothelial cells, and thus leading to pathology in DENV infections (Falconar, 1997; Lin et al., 2005; Cheng et al., 2009; Liu et al., 2011).

Based on these evidence, we had constructed a modified DENV2 sNS1 with deletion of epitopes mimicking autoantigens (ΔNS1), and found that prior immunization with either ΔNS1 or full-length NS1 in AG6 mice significantly helped to neutralize NS1 antigenemia and block the transmission of virus to mosquitoes. Notably, unlike antibodies elicited to full-length NS1, antibodies generated against ΔNS1 did not cross-react with human endothelial cells or human primary platelets. And after infection, viremia in mice immunized with modified-NS1 (ΔNS1) was significantly lower than those of mock or full-length NS1 immunized mice (Liu J. et al., 2016). In agreement with these results, mice immunized with ΔNS1 showed less vascular leakage and higher survival rate in AG6 mice after DENV-2 challenge, compared to mice that were immunized with full-length NS1 or PBS. Collectively, through antibodies specific to NS1 proteins that have been removed cross-reactive epitopes to autoantigens, we had successfully overcome the adverse effect of NS1 antibodies, blocked virus transmission, inhibited virus replication and protected mice from lethal diseases. Importantly, antibodies to NS1 do not have any risk of inducing ADE.

All above suggest that NS1 is an important target for blocking DENV/ZIKV human-mosquito transmission, and the modified NS1 elicits protective immunity toward ZIKV/DENV infection. Inclusion of suitable form of NS1 in human vaccine might offer a better chance for defeating DENV and ZIKV epidemics.

## Special Concerns in the Development of ZIKV and DENV Vaccines

Because ZIKV and DENV have different pathogenic characteristics and tropism, special issues should be considered during the development of vaccines to these viruses, especially for prophylactic ZIKV vaccines which are currently under development by several research groups (Richner et al., 2017b; Tebas et al., 2017; Medina et al., 2018; Modjarrad et al., 2018; Zou et al., 2018).

At the early stage of vaccine development for these two viruses, the primary aims are to inhibit virus replication in vaccinated animals, and to reduce mortality in lethal infection murine models. However, ZIKV has the ability to infect and replicate in reproductive systems, it can disseminate through sexual and vertical transmission, and it is associated with increased incidence of microcephaly in fetus. It is still unclear what level of viral load is enough to cause vertical or sexual transmission, or induce inflammation in these tissues and placenta, or damage testicle, or contribute to fetal microcephaly. According to the literature, viral clearance might be more difficult in these organs comparing to others (Barzon et al., 2016; Govero et al., 2016; Mansuy et al., 2016; Prisant et al., 2016; Miner and Diamond, 2017). Therefore, during ZIKV vaccine development, specified criteria for protective efficacies should include reduced neuropathology in adult mice, inhibited vertical transmission to fetus in pregnant animals, and decreased damages to reproductive systems. In contrast, evaluation of DENV vaccine in disease models mainly includes blockage of vascular leakage or hemorrhagic manifestations in mouse model.

Correspondingly, in clinical trial, severe dengue (DHS/DSS), virologically confirmed dengue, and hospitalization were the three major parameters to evaluate DENV vaccine candidates (Hadinegoro et al., 2015; Biswal et al., 2019). In addition to these three parameters, for the evaluation of ZIKV vaccine, other parameters might have to be included, such as neurological diseases (Guillain-Barre syndrome, fetal brain development abnormality).

To fulfill the criteria above, ZIKV vaccine strategy should also be modified, since the characteristics and type of protective immune responses in different organs are not the same. For example, the access of T cells to maternal-fetal interface was limited during pregnancy (Nancy et al., 2012). It has been found that weaker T cell responses were elicited by a live attenuated ZIKV vaccine during pregnancy, comparing to that in non-pregnant mice. In addition, higher neutralizing antibody titers were required to protect pregnant mice and block vertical transmission of ZIKV (Shan et al., 2019). Such evidence warrants a vaccine with higher potency in eliciting T cell and B cell immunity. In another study, it is observed that CD4 + T cell mediated antibody response, but not CD8 + T cells, was essential for viral clearance in intravaginal infection model. Though further investigations are necessary, this might imply the importance of CD4 + T cell immunity in blocking sexual transmission, which should also be considered during vaccine design (Elong Ngono et al., 2019).

## A One-Two Punch Strategy

**FIGURE 3:**
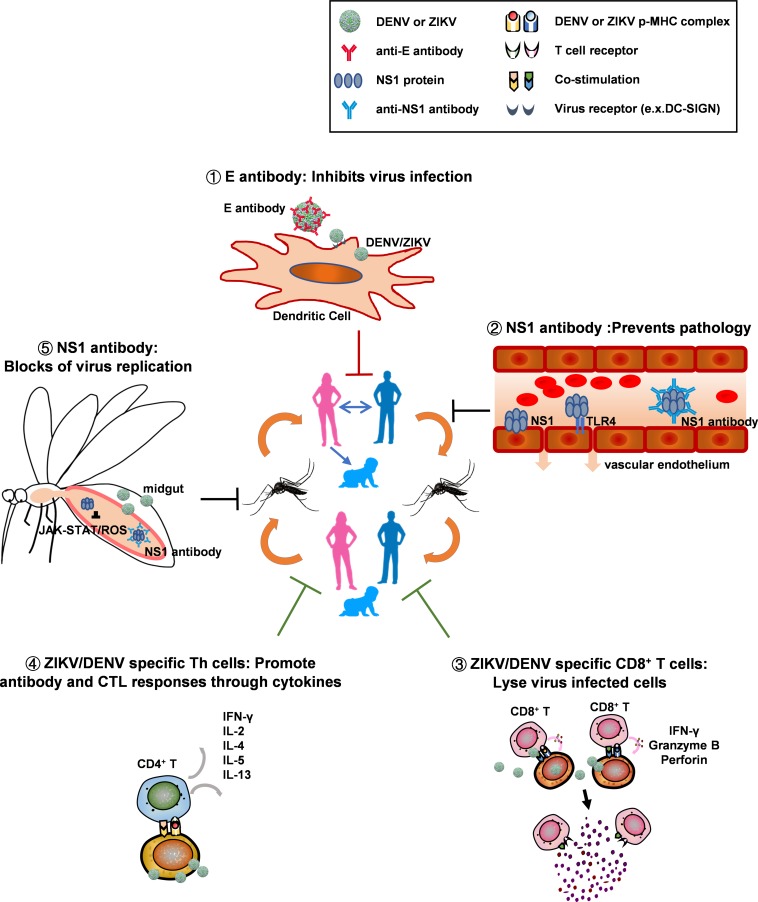
Urban cycle of dengue and Zika virus transmission can be blocked at multiple steps by vaccine elicited immune responses. Both dengue and Zika viruses are transmitted between human through mosquitoes in urban areas, where prevention measures can be applied. By human immunization with universal or pentavalent vaccines that elicit both T cell and B cell responses, especially those that also target NS1antigens, viral transmission in the urban cycle may be blocked and viral diseases can be controlled at multiple steps: (1) Virus transmission to humans and virus replication *in vivo* are inhibited by broadly neutralizing antibodies specific for envelope proteins; these antibodies block virus attachment and virus entry in dendritic cells or other target cells such as monocytes and macrophages; (2) Antibodies specific for NS1 proteins; these can reduce virus induced pathology in human through preventing NS1 hexamers attachment to endothelial cells, because the attachment may trigger the degradation of endothelial glycocalyx components or activate TLR4 pathway to produce vasoactive cytokines, both of which will increase endothelium permeability; (3) Virus replication in human is inhibited by functional CD8 + T cells. Vaccine induced memory CD8 + T cells can be activated by infected cells that present DENV or ZIKV peptides in the context of MHC-I molecules, and activated CD8 + T cells directly lyse infected cells by secreting granzyme B and perforin; (4) Both CD8 + T cell and antibody-producing B cell responses are facilitated by activated CD4 + T helper (Th) cells that secret cytokines such as IFN-γ, IL-2,IL-4, IL-5, or IL-13; (5) Viral transmission to mosquitoes is inhibited by NS1 antibodies. Mosquitoes acquire both NS1 protein and virus particles during a blood meal from acutely infected patients. The NS1 antigenemia could help virus to replicate in the midgut of mosquitoes, by antagonizing mosquito immunity such as JAK-STAT and ROS pathways. The abundant NS1 antibodies in human plasma of vaccine recipients may bind to NS1 protein and thus neutralize the antagonistic effect of NS1 to mosquito immunity, resulting in restriction of virus propagation in the midgut of mosquitoes.

After it is discovered for 70 years, we are still searching for better approaches to defeat dengue viruses. The sudden outbreaks of Zika virus has brought additional difficulties to solve the dengue problem, because the pre-existing ZIKV antibodies have the potential to enhance DENV infection, and antibodies elicited by dengue vaccines also have the potential to augment ZIKV infection. However, as for any other scientific challenges before, the large global outbreak of ZIKV has brought in extensive investigations on flavivirus virology, immunology, vaccinology, vector biology, and new tools to counteract the diseases. To combat mosquito-borne viruses, a one-two punch strategy that combines human preventive vaccine and vector blockade to cut transmission cycles at multiple steps may be necessary (as Figure 3 shows). Compared with other approaches for vector blockade, active immunization with NS1/ΔNS1 in human, is more feasible and has less spillover effect on ecology. Application of B cell vaccines producing broadly neutralizing antibodies may efficiently block virus infection in human and inhibit both vertical and sexual transmission of ZIKV. In addition, T cell vaccine can act in synergy with B cell vaccine to elicit functional T cell responses that inhibit virus replication in human, aiding to protect human from severe disease. At a minimal, incorporating NS1 within both B cell and T cell vaccines will likely to help overcoming the effect of ADE in human, blocking transmission to mosquitos, and providing opportunities for total disease control. However, further experimental evaluation on the feasibility, efficiency and safety of these approaches are necessary.

## Summary

We have reviewed the existing knowledge on epidemiology, molecular virology, and vaccine development of DENV and ZIKV; and updated our understanding on protective immunity against DENV and ZIKV. We have also discussed recent discoveries on the functions of flavivirus NS1 in viral pathogenesis and transmission. Based on such knowledge, we proposed a potential one-two punch strategy that overcomes ADE and defeats Dengue and Zika viruses through a combination of vaccines and vector blockade.

## Author Contributions

XJ conceived, drafted, and wrote the manuscript. JS drafted and wrote the manuscript. ZZ assisted to make the tables and drew the figure. SD and GC modified the manuscript, assisted to write, and amended the sections on NS1.

## Conflict of Interest

The authors declare that the research was conducted in the absence of any commercial or financial relationships that could be construed as a potential conflict of interest. The handling editor declared a shared affiliation, though no other collaboration, with one of the authors, XJ, at the time of review.
